# Spiritual and Emotional Care Among Clergy as First Responder–Victims in Puerto Rico: A Longitudinal Qualitative Study

**DOI:** 10.1007/s10943-024-02165-7

**Published:** 2024-11-01

**Authors:** Julianne Bryant, Melanie Nyhof, Michael W. Hassler, Jennifer Abe, Anthony Vives De León

**Affiliations:** 1 Department of Modern Languages, Biola University, 13800 Biola Avenue, La Mirada, CA 90639 USA; 2 Department of Psychological Science, Carthage College, Kenosha, WI USA; 3 Department of Social Work, Alvernia University, Reading, PA USA; 4 Department of Psychological Science, Loyola Marymount University, Los Angeles, CA USA; 5 San Juan Integrated Municipal Education System, San Juan, Puerto Rico USA

**Keywords:** Spiritual and emotional care, Puerto rico, Clergy, Chaplains, First responder, Disasters

## Abstract

A longitudinal qualitative study was conducted to explore the experiences of church leaders (10 priests, pastors, and pastors’ wives) who provided disaster spiritual/emotional care (DSEC) to the island of Puerto Rico during a period of intense and repeated crises from 2017 to 2022. Utilizing a narrative inquiry approach, 18 in-depth interviews were conducted and analyzed. Findings indicated that the participants engaged in psychological, social, and religious coping strategies to actively cope with the stress and trauma of being first responder rescuer/victims. Regional, cultural and contextual factors are considered in an effort to understand and enhance services to populations where disaster is the new normal.

## Introduction

There has been a five-fold increase in natural disasters over the past 50 years (Dunne, 2021). Consequently, disaster-related trauma has received much attention over the last several decades. A growing body of research has demonstrated the benefits of religious or spiritual emotional care in coping with disaster trauma (Almazan et al., 2019; Arkin et al., 2024; Aten et al., 2019; Chan & Rhodes, 2013; Cook et al., 2013; Davis et al., 2019; Feder et al., 2016; Lei & Wenhua, 2023; McElroy-Hetzel et al., 2018; Park, 2016; Smith et al., 2000) leading to the development of many resources for first responders (Aten, 2012; Aten & Boan, 2016; Aten et al., 2013, 2014, 2015; Brymer et al., 2006a, 2006b; Koenig, 2006; Massey, 2006; Roberts & Ashley, 2017; Schruba et al., 2018b). Clergy, by virtue of their profession, often serve as first responders in these recovery efforts. Several studies have encouraged collaboration between local churches and mental health care service providers (Arkin et al., 2024; Aten et al., 2010, 2013; Curtis et al., 2017; Entwistle et al., 2018; Guthrie & Stickley, 2008; Sun et al., 2019). However, few studies have focused on the specific experience of clergy as Disaster Spiritual/Emotional Care (DSEC) practitioners. Therefore, the purpose of this longitudinal qualitative study was to explore the spiritual and emotional care of a small group of local clergy serving as DSEC practitioners in Puerto Rico during a period of sustained crises from 2017 to 2022.

### Background and Context

On September 6, 2017, Hurricane Irma skirted the northern coast of Puerto Rico killing 4 people and leaving 2/3 of the island without power and 34% of the population was without access to clean water. When on September 20, 2017, Hurricane María, recorded as fluctuating between a category 4 and 5, hit the southeastern coast of the island and traveled across the entire region, 60,000–80,000 residents who lost power during Irma still had not regained service. María hit the island at 6:15 AM with tornado winds of between 130–200 miles an hour and lingered for nearly 30 h, wiping out the entire power grid. Approximately 70,000 homes were destroyed and losses are estimated to cost close to $90 billion. In the days following the hurricane, the people had to endure intense flooding, which rose to between four and nine and a half feet in some areas (Meyer, 2017; Staletovich, 2018). The death toll, although not easy to assess, has been estimated to be between 1139 (Santos-Lozada, 2018) and 4645 (Sutter & Santiago, 2018). Internationally, it is recorded as the sixth fastest intensifying, the third costliest, and the island’s most destructive hurricane (Staletovich, 2018). Consequently, in the year following the storm, approximately 130,000 residents—almost 4% of the island’s population—migrated to the US mainland, abandoning their damaged homes and lost jobs, and leaving behind extended family, community, and their former livelihood (Sanchez, 2022).

In the summer of 2019, the island had not yet completely recovered from the hurricane when political crisis complicated their recovery efforts even more. After several political leaders were arrested for corruption, a chat was leaked that implicated the governor for inappropriate behavior. Under the threat of political trial and pressured by two weeks of constant protests outside his government residence, the governor announced his resignation (Valdez, 2019). Then in January 2020, as the Commonwealth was working diligently to recover and regroup from the turmoil, the island was hit again with natural disasters, a multitude of earthquakes that killed one, injured several more, and left thousands without shelter or electricity once again. By mid-January, more than 8,000 people were homeless, with an additional 40,000 camping outside their homes in fear that they were not secure shelters as the aftershocks continued for months (Hanna et al., 2020).

In March 2020, when the realities of COVID 19 hit the island, the government implemented conservative measures, including a strict curfew, a mandatory lockdown, a mask mandate, and specific regulatory sanitation procedures (Acevedo, 2021). Yet, these protections did not prevent them from experiencing another extreme financial hardship with high rates of unemployment (Perez Semanaz, 2020), 30,000 lost jobs, and the closure of 1400 businesses. Once again, the people were mourning for “lost homes, jobs, businesses or loved ones” (Coto, 2021). Then in September of 2022, almost five years to the day that Hurricane Maria hit the island, Hurricane Fiona made landfall in an area where 3,000 homes were still covered with blue tarps from the damage of Hurricane Maria. In the south and central mountain regions the winds reached 160 miles per hour and the damage from the rain and consequent flooding exceeded that experienced after Maria, necessitating the temporary shelter of more than 2000 people across the island (Sanchez, 2022). The government response to the years of disaster in Puerto Rico has been limited and less than adequate, opening the door for the church to step in and provide not only immediate physical relief, but spiritual and emotional support during a period of extended crises (Lee & Ríos, 2018; Ríos, 2018; Rodríguez-Díaz, 2018).

### Spiritual and Emotional Care in the Aftermath of Disasters

The experience of natural disaster is not unique to Puerto Rico, especially in recent years. According to the United Nations Environment Programme (2018), since the beginning of the twenty-first century there have been more than 25,000 disasters globally, affecting more than two billion people and necessitating the mobilization of international relief teams. Economically thriving countries receive the resources necessary to recover from natural disasters, whereas poorer nations struggle significantly in the recovery process—such is the case for Puerto Rico (Cabán, 2021; Espinoza Vasquez & Oltmann, 2023; Rodríguez-Díaz, 2018). These disadvantages also lead to a higher risk of posttraumatic stress disorder (PTSD) (Roysircar & O’Grady, 2022). Additionally, communities that have suffered from prior catastrophic disasters are more susceptible to current disaster stress caused by both the financial and material impact (Friis et al., 2023).

Religion has been shown to provide a forward focus, beyond one’s current circumstances and, thus, create an empowering and unifying function in the context of crisis (Emmons, 2005). Various studies have demonstrated that those who are religious tend to demonstrate more positive coping and higher rates of resiliency following stressful or traumatic events (Ano & Vasconcelles, 2005; Darling et al., 2004; Fischer et al., 2006; Guthrie & Stickley, 2008; Koenig et al., 2001; Schuster et al., 2001; Spence et al., 2007; Weaver et al., 2003), some even experiencing posttraumatic growth (Exline & Martin, 2005; Murray-Swank & Pargament, 2011; Pargament, 2007). This understanding has informed the scientific study of religion in dealing with the aftermath of a natural disaster, an emerging and growing field of inquiry. According to Aten et al. (2015), disaster trauma is different than other forms of trauma because it affects the entire community. Thus, over the years, many resources have been developed to facilitate the integration of religious and spiritual elements into disaster relief efforts (Aten, 2012; Aten & Boan, 2016; Aten et al., 2013, 2014, 2015; Brymer et al., 2006a, 2006b; Koenig, 2006; Massey, 2006; Roberts & Ashley, 2017; Schruba et al., 2018b). Disaster spiritual and emotional care (DSEC) is defined as “any religion/spiritual (R/S) psychological intervention provided to disaster survivors in order to (a) mitigate their psychological and R/S distress and (b) promote their mental and R/S health and well-being” (Schruba et al., 2018a, p. 57). Several studies have demonstrated the benefits of positive religious and spiritual care among disaster survivors (Almazan et al., 2019; Arkin et al., 2024; Aten et al., 2019; Chan & Rhodes, 2013; Cook et al., 2013; Davis et al., 2019; Feder et al., 2016; Lei & Wenhua, 2023; McElroy-Hetzel et al., 2018; Park, 2016; Smith et al., 2000). Thus, collaboration between mental health providers and churches has been encouraged (Arkin et al., 2024; Aten et al., 2010, 2013; Curtis et al., 2017; Entwistle et al., 2018; Guthrie & Stickley, 2008; Sun et al., 2019).

### Clergy as First Responders

First responders, such as firefighters, emergency medical technicians, or police officers, are those who are the first present at a trauma or disaster site and are the first to attend to the victims. Although the need for first responders has increased due to the frequency of natural disasters (Dunne, 2021; UN Environmental Programme, 2018), communities of first responders are studied less often than the victims they serve. Those studies that have been conducted indicate a higher incidence of posttraumatic stress and depression among this population (Kleim & Westphaul, 2011; Neria et al., 2011; Sakuma et al., 2015; Zhang et al., 2021). Self-care, social support, and perceptions of belonging have been associated with resiliency and lower levels of PTSD symptoms and burnout (Chen et al., 2022; Haslam & Mallon, 2003; Lanza et al., 2018; Prati & Pietrantani, 2010; Stanley et al., 2019). Yet, Lanza et al. (2018) suggest that different categories of first responders, like firefighters or clergy, have different needs, so treatment should be tailored accordingly.

During the recovery efforts of Hurricanes Andrew, Katrina, and Rita, a new category of first responder was identified, that of the “simultaneous rescuer-victim,” to reflect that they and their families are victims of the disaster to which they are responding (Flannery, 2015). Those who are victims and responders often feel torn between attending to the needs of their own family and to those of the community (Lanza et al., 2018). Although there is not much research on treatment for this population, Castellano and Plionis (2006) found that the success of the treatment technique depends on the stage of recovery and the context of the event. For example, during the stabilization stage of ground zero, three interventions proved beneficial for the newly trained team members: (1) routine psychological check-ups, (2) ongoing spiritual leadership, and (3) consistent access to media for updates. However, during the triage phase, it was necessary to assess job performance on an hourly basis and give team members the opportunity to regroup.

The surge in natural disasters world-wide has increased the need for qualified humanitarian aid workers, a specific type of first responder. Many are flown in from outside the region to provide immediate aid. Several studies have indicated that there is a greater incidence of mental health issues like anxiety, depression, PTSD, and burnout among this population (Ager et al., 2012; Cameron et al., 2024; Eriksson et al., 2001; Jachens et al., 2018; Lopes Cardozo et al., 2013; Strohmeier et al., 2018). However, Ager et al. (2012) found that higher levels of social support and team cohesion reduced the incidence of burnout. Likewise, DePaul and Bikos (2015) found that a higher degree of organizational support was associated with increased psychological well-being. One reason for the higher rate of stress and burnout among international workers may be that they are in an unfamiliar setting. National workers, on the other hand, may have even higher rates of distress in cases when they are affected by the humanitarian crisis themselves, just like the simultaneous rescuer-victim, which might also increase the risk of suffering from PTSD, anxiety, or other mental health conditions (Cameron et al., 2024).

There are very few studies that have considered national humanitarian workers. One study examined locally recruited relief workers, both nationals and expatriates, and reported higher levels of depression, anxiety, and PTSD from exposure to traumatic events. However, social support was found to be a protective factor for anxiety, and team cohesion, a buffer against burnout (Eriksson et al., 2013). Another study among Kosovar Albanian workers found that the nationals experienced more exposure to trauma and more elevated levels of anxiety, depression, and PTSD when compared to the expatriate workers (Lopes Cordozo et al., 2005). Captari et al. (2018) studied religious coping among Filipino humanitarian aid workers who had a long-term involvement and personal connection to the disaster. Results suggest that negative religious coping was associated with increased burnout. However, although previous studies did find that positive religious coping promoted emotional well-being (Ano & Vasconcelles, 2005), this particular study did not find that positive religious coping helped to mitigate burnout among this population (Captari et al., 2018). The authors postulate that this could be a result of sample characteristics like culturally specific nuances not captured by the Brief RCOPE or even conjectured that perhaps religious coping is not helpful in reducing symptoms of burnout. This suggests that more research is needed among other cultures and within different contexts.

Chaplains are a specially trained class of first responders who provide spiritual care intervention services in critical environments alongside other first responders like paramedics, hospital personnel, the military, and the police (Layson et al., 2023). Several studies have documented the importance of chaplain services and specialized training as they provide holistic care and psychological first aid, not only to the public, but in support of the other members of the emergency team (Carey & Hodgson, 2018; Childers, 2012; Layson et al., 2023; Tunks Leach et al., 2022, 2023, 2024). However, due to their role on the front line, they are also at a higher risk for secondary traumatic stress and burnout (Harris et al., 2023; Hotchkiss & Lesher, 2018; Kelly et al., 2024). Mindful self-care, social support, and a sense of purpose have been found to be protective factors for burnout (Hotchkiss & Leshner, 2018; Kelly et al., 2024). In a study that explored the role of chaplains who served in both the AIDS and COVID-19 pandemics, chaplains found that having served in a prior pandemic helped them develop coping strategies that mitigated burnout for them—such things as support networks, humor and hobbies (Kelly et al., 2024). Another study explored the impact of deployment on psychosocial and health characteristics among those serving in the national guard (Besterman-Dahan et al., 2012). They found that those who had been deployed presented significantly higher levels of resilience. Additionally, although levels of negative religious coping were low overall among both groups, they were slightly higher among those that were deployed, which may have been due to the double burden of experiencing trauma while providing support and care to service members.

Like chaplains, local clergy offer support to their congregations throughout many life crises, including natural disasters. Most pastors or priests are prepared to provide spiritual and emotional support for personal tragedies, yet few are properly trained to handle large-scale natural disasters (Cain & Barthelemy, 2008; Darling et al., 2004). Following the November, 1985 floods in West Virginia, Bradfield et al. (1989) found that clergy were key first responders in ministering to the physical, emotional, organizational, and spiritual needs of the community. However, they also experienced symptoms of posttraumatic stress disorder such as fatigue, feelings of guilt, and burnout during the months following the disaster. Similarly, African American pastors, one year after Hurricane Katrina, reported feeling overwhelmed with the number of individuals seeking emotional support, even among those who had advanced degrees and training in Christian counseling. This was further intensified in cases where the pastors had also lost their homes or had been victims of significant damage (Aten et al., 2010).

### The Current Study

The purpose of this current study was to examine the experience of a small group of local clergy as DSEC practitioners in Puerto Rico throughout a sustained period of crises from 2017 to 2022. In the absence of governmental and national relief, the church was at the center of the recovery efforts, which involved the clergy as first responder/victims. The series of events that took place created a period of extended crisis for everyone on the island. Recovery from one crisis did not occur before another began, creating a unique situation for pastors and priests as DSEC practitioners.

In spite of the growing field of inquiry in religion and spirituality in the care of disaster survivors, the studies examining disaster spiritual and emotional care (DSEC) in practice are very limited. Even fewer are investigations among clergy, and none have focused on clergy as “simultaneous rescuer-victims.” Moreover, many of the studies have investigated survivors’ recent reactions after a disaster, not in a context of ongoing sustained crises as in the case of the clergy in Puerto Rico. Furthermore, with the increase in disasters world-wide there is a need for more studies that incorporate a distinct cultural perspective to add to the growing field of disaster spiritual and emotional care in practice.

For these reasons, this longitudinal qualitative study examined the experience of a small group of local clergy in Puerto Rico, understanding that their experiences as DSEC practitioners may have been uniquely influenced by their experience as both victims and first responders. The study explored (a) the cultural, geographic, and familial influences on their experiences as DSEC practitioners in different types of crises, (b) the effects of sustained ongoing crises upon serving as DSEC practitioners, and (c) the ways they experienced spiritual and emotional care throughout their recovery efforts.

## Methods

### Participants

Following Biola University’s Protection of Human Rights in Research Committee’s (PHRRC) approval (Protocol # SS19-003_JB), 18 in-depth interviews were conducted with 10 participants between June 2019 and September 2022, all pastors, priests or pastors’ wives; a total of 5 women and 5 men between the ages of 39 and 60 at the onset of the study. A purposeful sample was drawn among pastors and priests from three Christian denominations (Iglesia Cristiana Discípulos de Cristo, las Catacumbas, the Roman Catholic Church) who were identified as being involved in recovery efforts on the island in the aftermath of Hurricane Maria. Initial contact took place over email and personal appointments were set up among those who responded indicating an interest in participating in the study. Demographic data for each participant are shared in Table 1.

**Table 1:** Demographic data for participants

Participant	Denomination	Role	Area	Age	Gender	Property loss/damage	Injury
P1	ICDC	Pastor	M	39	M	X	
P2	ICDC	PW	C	37	F	X	
P3	ICDC	Pastor	C	42	M		
P4	ICDC	PW	C	39	F		
P5	ICDC	Pastor	C	47	F	X	
P6	Catacumba	Pastor	C	60	M	X	X
P7	Catacumba	Pastor	C	44	M		
P8	Catacumba	PW	C	44	F		
P9	Catholic	Priest	M	48	M	X	
P10	Catholic	Priest	C	58	M		

P: participant, ICDC: Iglesia Cristiana Discípulos de Cristo, PW: pastor’s wife, M: metropolitan area, C: Campo (rural), M: male, F: female

### Data Collection

In accordance with the PHRRC protocol, each participant completed a consent form to participate in the study before engaging in a 30–60-min face-to-face interview at their church. Data collection began at the onset of the political crisis in the summer of 2019 and ended when Hurricane Fiona hit the island in September 2022. Follow up interviews were conducted either in person or via Zoom. A narrative inquiry approach was utilized to collect participant stories (Clandinin & Connelly, 2000; Creswell & Poth, 2018) based on the primary investigator’s (PI) desire to give voice to individual experiences and gain insight “by listening to and reflecting together with participants on their narratives” (Bruce, 2008, p. 325). Thus, the participants were asked to share their experience of the crisis, describe how they participated in the relief efforts and how they experienced spiritual and emotional care during each crisis. All interviews were audio recorded and transcribed verbatim. The PI also attended church services at each site and recorded field notes before, during and after each interview/field visit, engaging in reflective practice throughout the data collection process.

### Data Analysis

Once transcribed, the PI listened to each interview and checked the transcripts for accuracy. Bilingual research assistants (RA) then translated all transcripts from Spanish into English for the non-Spanish-speaking members of the research team. The translations were reviewed for accuracy by the PI. The research team engaged in multiple rounds of reading the transcripts and taking notes to develop the first set of codes. By identifying recurring themes, initial coding was done to identify data that answered the research questions or offered new insights. Spanish transcripts were then uploaded to Dedoose Version 9.0.86 (Salmona & Lieber, 2020) for two more rounds of coding utilizing the software features to organize emerging codes into families as we moved to more abstract levels of analysis. Inductive line-by-line coding was conducted by the PI and one bilingual RA. During this phase of analysis, we conducted weekly team meetings to discuss the emerging codes. To prepare for these meetings, MN read the English translations of the transcripts being discussed. Once all the transcripts were coded, the PI in consultation with MN expanded and organized the original categories into overarching themes to prepare for selective coding in which each transcript was again coded by the PI and a different RA. During this phase, codes were authenticated, matched and condensed into three final themes that built a storyline explaining the interconnecting categories.

### Trustworthiness

Various methods consistent with Creswell and Poth (2018) were engaged throughout the data analysis process in order to establish trustworthiness and credibility. During the first phase of coding, the team met weekly to ensure consistency in interpreting the codes and to answer any questions that would arise. Each transcript was individually coded by three coders. Member checking occurred when the RAs acted as second coder, verifying the codes that had already been logged. Each RA kept a reflexive journal for this part of the analysis as they interpreted the interview data and assigned the newly organized codes. This allowed them to be critically reflexive about their positionalities as it related to the research participants and the data they were interacting with. Additionally, peer review occurred when the PI met individually with each RA after selective coding and the codes were merged to check for accuracy. Any discrepancies were discussed and they had to arrive at 100% agreement or else the issue was taken to the group meeting for discussion. Only the agreed upon set of codes were maintained for analysis; the other sets were discarded.

## Results

Although all participants mentioned symptoms consistent with PTSD or burnout (e.g. fatigue, exhaustion) in the early months following Hurricanes Irma and Maria, one of the themes that was mentioned repeatedly was the stark difference in the storm and recovery experience for those living in regions outside of the metropolitan area. While the stress of the recovery efforts in the San Juan area dissipated within a couple months, for the internal parts of the island, the intensity lingered for much longer.

My family is from the metro area. To know that they had water after only one month. In my home after three months, we still had no water. In January they had electricity and in my home in January we still had no electricity. To get up here and get across what I had to cross, that whole journey to get home, to know that some people, right?, the difference. The big difference in living in other places. They didn’t have that daily journey and when they got home, they had electricity in their house. But for us, it wasn’t just the intensity of the drive, but also to get home at 7:00 or 8:00 at night just to turn on the generator so you could have some power because we weren’t able to rest. We, my family, in my house we lived that difference that in one part of the island they had returned to life as normal while we were still living like this (points to the window where you can hear very noisy traffic passing by). Just driving so…just driving was a daily issue. It was a daily issue. And that difference was substantial. And we lived it, you know? Or we saw it, how everything was working and we were still in that fatigue, in that unending stage, in that rush because there were people who still had nothing, who would return but had nothing. They had jobs but they had no way of getting there. There was no way to get there. Up here there were roads that were literally not opened for months. One of the highways, the 143, is still closed because it’s gone. It collapsed along with half the mountain. So, someone who lives 10 minutes from their job still has to go so far out of their way to get there because the road’s not there anymore, because the highway’s not there, and that’s real. (P3)

[See Appendix 1, Quote 1]

As this participant pointed out, the experience for those outside of the metropolitan area was much more difficult. In terms of the difference between crises, the participants indicated that the overall impact of Hurricane Maria and the global pandemic were the most difficult to navigate due to the fact that the entire island was implicated. The other disasters, although devastating for the areas affected, did not have such a universal effect, enabling the unaffected areas to mobilize quickly with more expertise and compassion due to the lessons learned while in recovery from Maria. During the political crisis, the participants were very much affected and this was evident in their focus during the interviews as well as in their sermons and prayers at church services. However, the outcome achieved by the governor’s resignation had a unifying and positive effect that seemed to reduce the significance of this crisis for the clergy when compared historically to the other disasters.

**Fig. 1:**
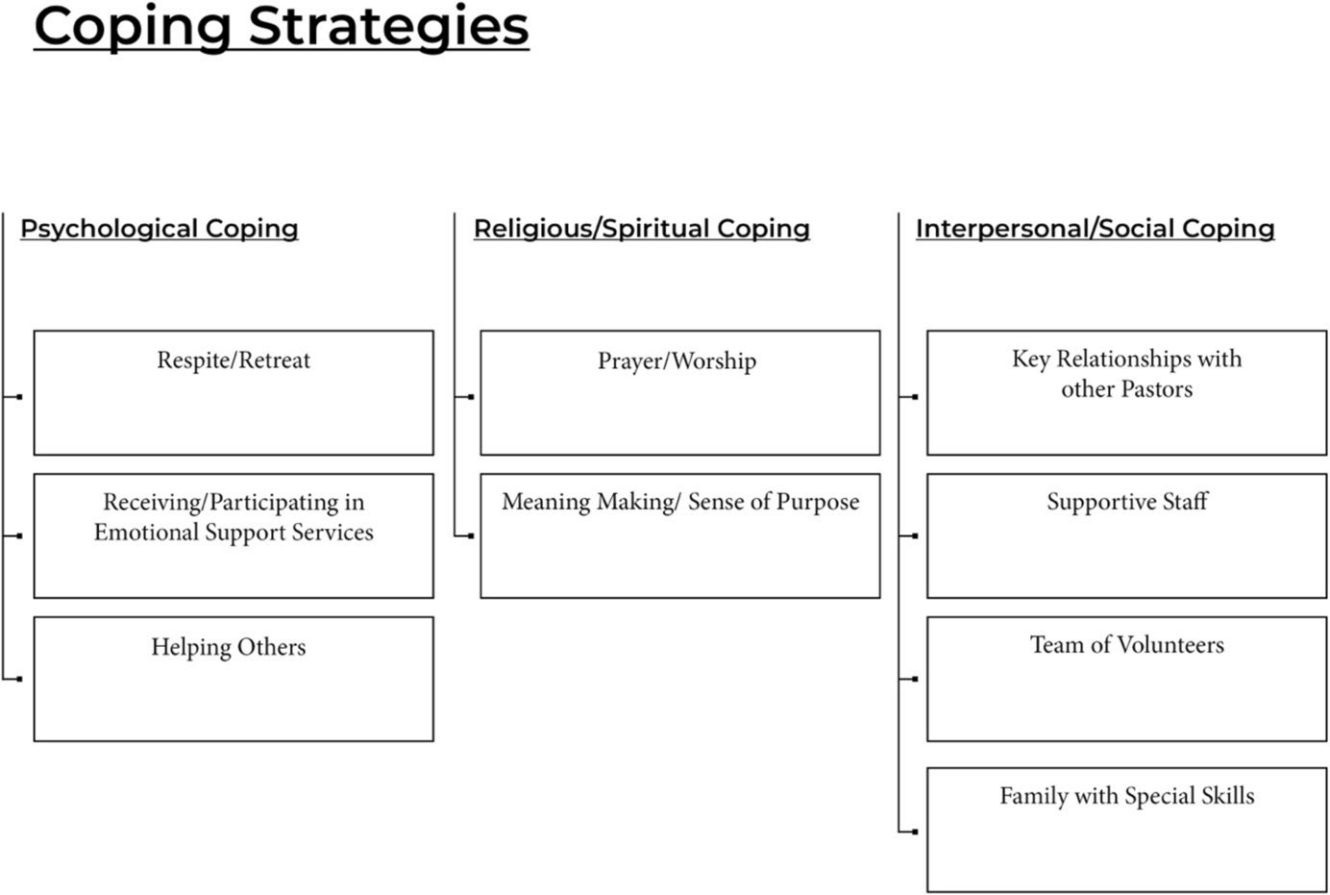
Active coping strategies

Beyond these differences, findings indicate that there were several active coping strategies utilized by the clergy in order to receive spiritual and emotional care in the midst of crises. These strategies were grouped into three primary categories: psychological coping, religious/spiritual coping, and interpersonal/social coping. Although these strategies spanned across all crises, the context of each crisis as well as the clergy influenced the strategies that were utilized. A summary of the strategies utilized can be found in Fig. 1.

### Psychological Coping

The participants employed various strategies and resources to cope psychologically with the tragedies that faced them: respite/retreat, participating in emotional support services, and helping others.

*Respite/Retreat* Due to the extent of damage from Hurricane Maria, in the aftermath, the clergy were working from sunrise to sunset seven days a week for months. It seemed as if there was no time to rest. However, the energy exerted was not sustainable and the participants realized they needed to get away, identifying that the only way possible was to leave the island.

We were still going form emergency to emergency. And then when in January I decided to get and…We have to literally leave the country because there was no way to disconnect ourselves without leaving…And I think those few times, that vacation in January helped us to breathe, helped us to recover, helped us to go out and get air. Because there comes a time that you’re not just tired, you start to erode. (P3)

[See Appendix 1, Quote 2]

We were able to have time to organize ourselves and learn different strategies to figure out what we’re going to do, how we’re going to do it, and we returned stronger to Puerto Rico. But if it weren’t for that. (P2) [See Appendix 1, Quote 3]

And then we left. And it was truly wonderful because we returned refreshed with a clearer vision of what to do, how to do it and new ideas unfolded….And perhaps after that, the next year, about six months later…we also got away. We had a three-day retreat with different pastor friends. (P8) [See Appendix 1, Quote 4]

These participants identified that their physical removal from the island was necessary to continue in the recovery effort, giving them a renewed strength and vision. For some pastors, getting away was part of their regular practice. For others it was a lesson learned through their experience after Hurricane Maria.

I also tend to take personal spiritual retreats; I go to a retreat house that is in the central mountain area and I spend those days resting, relaxing y connecting with God. (P9)

[See Appendix 1, Quote 5]

Obviously, we have been pastoring for 22 years. I think that experience has helped us understand the importance of resting. So, as soon as other leaders stepped in, we started to take the time we needed. We started to protect that vacation time in order to avoid burnout because in the past that happened to us. (P7) [See Appendix 1, Quote 6]

This pastor indicated that taking breaks is only possible when there are sufficient leaders present to continue to serve in the pastors’ absence. Pastors of smaller congregations, lacking in an extended leadership team, cannot regularly take advantage of this form of spiritual and emotional care. These breaks were crucial to the continued functioning of this group of clergy and their wives. A more extended break was necessary for one of the pastors of a smaller rural congregation who, during COVID-19 did step down from ministry for several months. He stated that the break was necessary to avoid burnout.

*Receiving/participating in emotional support services* Being clergy in an extended period of crises highlighted the necessity for many participants to identify their limitations and ask for help. Some did it proactively, others reactively. This help took the form of individual counseling or psychotherapy, group counseling, marriage counseling, or church-organized or denominational support groups with other leaders or pastors.

Through the denomination we received help. We had space. And personally, we had time with health professionals because it’s necessary, because you realize that it’s there. With everything that’s happened I already mentioned that it was a year, that after a year was the first time I cried. (P3) [See Appendix 1, Quote 7]

For this participant, the services were provided through the denomination in the aftermath of the hurricane. Others shared participating in group counseling with other pastors.

And that plan, in April of this year, all the pastors, they all stayed at the Hyatt in Manatí and they brought in outside resources in order to teach the pastors how to work with depression, how to work through difficult moments of crisis and they provided these services from Wednesday to Wednesday so the pastors of this area could stay there, receive the resources and process their emotions. (P5) [See Appendix 1, Quote 8]

One priest from an area where the earthquake tremors were very strong, described his response to the trauma as well as his need to seek psychological support.

And I never experienced an earthquake like this one. Those tremors really affected me and thank God I had to go to see a psychologist for a while. We also need the help of psychologists. And it’s helped me move forward with respect to my personal issues. (P10) [See Appendix 1, Quote 9]

And for others, this realization happened after years of constant crises.

It was a very rough time, intense, that at a certain time I said, “You know, I can’t do it anymore, I’m tired.” And we even began to receive therapy. (P 4)

[See Appendix 1, Quote 10]

*Helping others as coping* Many participants mentioned that the act of participating actively in the relief efforts, especially in response to Hurricane Maria, actually helped them cope and provided them an outward focus on others versus their own situation.

But God strengthens you because it’s possible that as soon as the need to serve arises you forget the rest. The fact that you’re tired, that you still haven’t recuperated from the hurricane. No, when you need to serve, when it’s a vocation of service, right?, well certainly you always serve the best you can and forget about the rest, you know? (P9)

[See Appendix 1, Quote 11]

One pastor whose home was completely flooded and who was displaced to his in-laws for several months, described working long days and not even being able to think about his own loss, stating that it didn’t necessarily hit him until the evening when he returned to his in-law’s residence instead of his own home. He was focused on his work and on the project of helping others.

Yes, the things we were doing. We had meetings every week with Christ Collaborative, you know? With the different churches we organized things and we learned about the different projects. So, my mind was distracted. (P1) [See Appendix 1, Quote 12]

### Religious/Spiritual Coping

As pastors and priests, religious/spiritual coping methods were part of their practice and participants specifically mentioned prayer, community worship and a deep sense of purpose or calling as a way to find meaning in their relief work.

*Prayer/worship as a source of comfort* The participants mentioned prayer as a grounding practice throughout all crises.

Well, obviously what helped me the most was prayer, you know? Daily prayer, reflection, taking the time to mediate, taking the time to turn off the cell phone and sometimes, if possible, take a walk on the beach. Daily mass also helped me a lot, you know? (P9) [See Appendix 1, Quote 13]

This priest discussed his individual practice of prayer. Group prayer was practiced frequently among the clergy in this study, as this participant describes.

I have priest friends who I meet with to drink coffee and share, but we end up praying for one another. And those times are good. (P5) [See Appendix 1, Quote 14]

*Meaning making/sense of purpose* Many participants mentioned that the role of the Church in the relief efforts after the hurricane changed the way they conceptualized their own role. Helping the community became a greater focus, as this participant describes.

I believe that I was focused as a pastor, leader…All my life I have taught leadership, leading in missions…I work, my work was centered on the Church, not on the community. My life changed completely from the hurricane and it has continued since then. I don’t think it’s going to change. María taught me that my main work was with the community, with the people, the Church is called to go to all the world…Well, in my case, it’s personal. My life changed completely. I mean, my focus now is to work within the community and the need of the people. (P6) [See Appendix 1, Quote 15]

For others, it confirmed the importance of their calling, providing a sense of meaning and purpose for their lives and connecting their work with God’s calling.

So, I feel that I have a vocation of service, right? And He has placed me in this vocation to serve the most needy, right?, through my job. Therefore, when I had to respond, when we had to respond, we did. (P9) [See Appendix 1, Quote 16]

### Interpersonal/Social Coping

Because the Puerto Rican culture values community, the typical coping strategy in the aftermath of Hurricane Maria was to work together. Many participants mentioned the increased sense of community that was engendered during that time. However, this was not the case during the pandemic. The complete isolation in the early months of the pandemic and the lack of outside help made it seem like more of a crisis than the hurricane.

But those first two months, they were a burden for me and my wife, my whole family…From outside, well, with the hurricane there was, there was more help from outside. Maybe because the pandemic affected everybody, I didn’t necessarily receive a call from somebody offering, “Come stay with us to take a break.” Or stuff like that. During the pandemic that didn’t happen. I think it’s because we were all in the same boat, right? (P7) [See Appendix 1, Quote 17]

The defining factor was the level of support within the church and the family that created a sustainable infrastructure. Participants mentioned key relationships with other pastors, supportive staff, teams of volunteers, and family with special skills as the primary social support that helped them cope with the crises.

*Key relationships with other pastors* One area of change in the Church in Puerto Rico was the formation of groups that bridged denominations. The grave necessities in the aftermath of the hurricane created the need for collaboration of all church leaders, regardless of denomination or affiliation. This created a space for key relationships within and outside of current networks to function as a primary area of social support for the clergy in their role as responder–victim. They strategized together, met regularly, combined resources, shared hardships and victories, and supported each other spiritually and emotionally. Some new friendships were formed, and bridges were created, especially between Catholic and Protestant denominations, who did not historically work together on the island. One pastor described the support she received from this network.

Throughout the time we have had a support group of pastors and priests that met regularly to vent. And that’s a blessing. So as a leader, I had that network of support. But not everyone has that. (P5) [See Appendix 1, Quote 18]

For some this network extended to churches and pastors on the US mainland. One pastor’s wife describes being cared for by a pastor that they visited in Texas to observe what was happening in the relief effort following Hurricane Harvey.

And even when we left, they treated us like, they pastored us, I mean, they embraced us, they took us to see the community, they allowed us to interview the community members there, the undocumented, but the church that was helping us was helping them. They wanted us to see outside of our circumstance. Then we went with our pastor friend to an event he was hosting. He wanted to take us out, to talk about other things as well. And we were with other friends from the Catacumba denomination and another organization that’s called Hunger, and that for us was enough to return with the motivation to keep working. (P2) [See Appendix 1, Quote 19]

These relationships built over times of crises became lasting friendships of support throughout these five years of instability. However, the pastors did mention that there seemed to be less support and communication during the pandemic as the whole world was affected. They had less time to communicate, less desire to spend even more time on social media than was required by their job, and there was less aid. Since the pandemic was not unique to Puerto Rico, they did not get as many calls from US-based support networks as they did during the other crises.

*Supportive staff.* In the aftermath of the initial disaster, a supportive staff was a crucial element of care for clergy. The only way they managed to complete the work was by leaning on each other. One priest describes a daily practice of spiritual and emotional support that helped them to process all that was happening to prepare them for the work they had before them.

Being conscious of that duality that we are rescuers but we are also victims. I think that’s very important, especially during the first two months that were the most chaotic. What did we do? Well, we dedicated an hour before we opened our services to pray and talk. I think that was very important. It was vital so that we could accomplish our work because nobody can give from an empty well. So, you have to fill up first before you give. And I think that dynamic helped all of us because people cried, people talked about what was happening in their homes. (P9) [See Appendix 1, Quote 20]

Another pastor described that the spiritual and emotional care came naturally within the context of the church, which helped them navigate the moments of despair brought on by their circumstances.

If there’s one area that I felt was never neglected it was in the spiritual and emotional. That’s the advantage of working with the church. You know, yes, the church, since the large majority were there. Clearly a fundamental part of the church is working in the spiritual area. There were moments of despair. (P6) [See Appendix 1, Quote 21]

*Team of volunteers* The participants also shared the importance of the many volunteers from within their churches as well as those that arrived from outside to aid them in their efforts. They felt as if God cared for them through those volunteers, giving them renewed energy and strength to endure.

And I, thank God that when I got to the church, the team was already there working. The needs were increasing, but thank God I am supported. I have a team, a whole team of parishioners that help me and sustain me to be able to endure. Without that team I think I would be helpless and it would be even more difficult to manage. (P10)

[Appendix 1, Quote 22]

But we saw the hand of God. In the church neighbors, the leaders who helped here. Yes, this was not an individual job. It was the work of many people, not one person. There were people that helped us. There were those who went the extra mile. There were missionaries that came for a week and those who offered to host them that gave us renewed strength and for that we give glory to God. There were many who came and if it hadn’t been for that, and you could see the hand of God through the people who came to lend a hand, to give us renewed energy, new air, and rest. So, it was a collective effort. (P3) [See Appendix 1, Quote 23]

*Family with special skills* The study participants frequently mentioned the support of family members as being key to their spiritual and emotional care. Among the Protestant pastors, their spouses were considered part of the pastoral team as described by this participant.

I have a marvelous wife, PI. (He laughs) A marvelous wife. She’s a social worker. So, we made a great team, and everything we were able to distribute from here was in an organized manner, very organized, to provide for people in need and to attend to those who clearly had nothing. (P3) [See Appendix 1, Quote 24]

However, nowhere did this topic come up more than with reference to the pandemic. When the pastors were separated physically from their broader support staff due to the social distancing regulations of the pandemic, the clergy were forced to rely more fully on their immediate families. Thus, those families with more members and broader skills were supported more than those with small children in rural areas with fewer resources. Some pastors expressed the benefit of having older children who were musicians with social media skills.

There’s a lack of awareness about COVID and everything else. And basically, well, we began to broadcast from my house. And I am blessed because my kids are musicians, my wife. So, we were running the whole technological aspect. (P7)

[See Appendix 1, Quote 25]

Another had a spouse that worked in public relations and brought a wealth of knowledge and experience to the newly defined role of home broadcasting.

Something that helped us is that P2, my wife, she’s worked in social media for several years and we have the equipment to record. For example, I’ve been using Zoom for like seven years. So, it wasn’t anything I had to learn how to do. (P1)

[See Appendix 1, Quote 26]

However, for pastoral families that did not have internal support systems or for churches with more limited resources based on the location, experiences during COVID were more intense.

It's difficult now. But it depends on your location. For example, I know in P3’s case, who you know, for him it became difficult because in his area there is a weak internet network, plus he had no equipment. And the church where [an employee’s] mother attends in Ciales, they weren’t doing anything besides what they could send via voice messages in WhatsApp. (P1) [See Appendix 1, Quote 27]

## Discussion

This study sought to examine the experience of a small group of pastors, pastors’ wives, and priests serving as DSEC practitioners in Puerto Rico across a series of five years of intense crises, from 2017–2022. Like other studies (Ager et al., 2012; Aten et al., 2010; Cameron et al., 2024; Eriksson et al., 2001; Jachens et al., 2018; Kleim & Westphaul, 2011; Lopes Cardozo et al., 2013; Neria et al., 2011; Sakuma et al., 2015; Strohmeier et al., 2018; Zhang et al., 2021), this group of first responders all experienced high levels of stress across this time, especially in the long months following Hurricane Maria and in the initial months of the global COVID-19 pandemic. However, the participants were able to use psychological, social and religious coping strategies to actively cope with their situation.

These findings are in line with previous studies that have suggested that those who are religious show more positive coping and higher rates of resiliency following a disaster (Almazan et al., 2019; Ano & Vasconcelles, 2005; Arkin et al., 2024; Aten et al., 2019; Darling et al., 2004; Davis et al., 2019; Fischer et al., 2006; Guthrie & Stickley, 2008; Koenig, 2006; Lei & Wenhua, 2023; Schuster et al., 2001; Spence et al., 2007; Weaver et al., 2003). Religion did provide a sense of purpose and a grounding focus through prayer (Emmons, 2005). Additionally, as with other studies (Ager et al., 2010; Chen et al., 2022; Haslam & Mallon, 2003; Hotchkiss & Leshner, 2018; Kelly et al., 2024; Lanza et al., 2018; Prati & Putrantani., 2010; Stanley et al., 2019), self-care, social support, team cohesion, and perceptions of belonging to a community served as a buffer for PTSD and burnout. All priests and pastors along with their wives continued to serve effectively in their communities, although one pastor who resided in a rural area did take a sabbatical during COVID-19.

Participants stated that serving during the COVID-19 pandemic presented more challenges than the other crises. One reason for this was that everyone had to serve on their own in isolation. After the hurricane they received more support and concern from outside the island, but during the pandemic, everyone was working in isolation. Those churches with fewer family members in areas with fewer resources were less able to shoulder the burden of responsibilities that came with pastoring a church at that time. Thus, although there were no denominational differences in how the clergy experienced the crises and served in the recovery efforts, there was a notable difference in the organizational support provided within the denominations. The Catholic church was working under a previously created infrastructure. They have an organization called Caritas (much like Catholic Charities on the mainland) which has a director and chapters across the island. This model made support easier to access and provide. The Protestant churches were moving toward a similar structure with the inter-denominational group that was formed during these crises, but it began in response to the disasters and was not fully developed throughout the island. A high degree of organizational support has been shown to increase psychological well-being (De Paul & Bikos, 2015). Having this level of support might have mitigated the need for P3 to step down from his position.

A consideration of certain aspects of the Puerto Rican culture helps to make sense of the findings in this study. Working together as a group and sharing resources, the way they came together after the hurricanes, during the political crisis, and in the midst of the earthquakes is fundamental to their cultural collectivist community roots (Carvalhaes et al., 2022; Espinoza Vasquez & Ottman, 2023). Thus, it would make sense that the stress of the pandemic seemed more intense than the other crises that they experienced in community. Additionally, the Puerto Rican people’s historical experience as a colony that has been oppressed has created for them a situation of agency in the face of crisis, of action in response to disaster, a call to serve others (Cabán, 2021; Petrun Sayers et al., 2023). Factors that lead to resiliency have been characterized as personal, familial and communal (Werner, 1995). The coping strategies that were utilized for the participants in this study can be characterized within those groups. Coping under adverse conditions has been said to lead to resilience. In the case of Puerto Rico, Rodríguez-Díaz (2018) characterizes it as “resistance,” stating, “The community response in Puerto Rico evidenced fundamental collective competencies that public health workers must nourish” (p. 31).

### Practical Implications

The natural disasters described in the present study are not a one and done. Rather, regular disasters are likely the new reality for residents of the island. Thus, it is important to continue to offer DSEC training to clergy. However, it is also important to recognize that these ministers will need ongoing care and support, as they are often both rescuers and victims simultaneously and must shoulder the burden of being first responders in the context of ongoing sustained crises. It is recommended to assess the level of need in all regions and provide support accordingly. Perhaps a team of visiting pastors can relieve those in need of more support, or can serve alongside those pastors for a designated period of time in order to lighten their load.

## Limitations of the Study and Future Directions

This study only includes data within a Christian context. The results may not extend to other Christian denominations or religions in Puerto Rico or beyond the Puerto Rican context. Church leaders from other religions would provide a richer analysis. Additionally, the purposeful sample of study participants, that of choosing study sites that had been involved in the recovery efforts, may determine a certain level of similarity in the findings. Perhaps if the participants would have been chosen randomly, different outcomes may have been identified. As mentioned earlier, many steps were implemented in the data analysis to reduce bias in the research findings, such as member checking, reflexive practice, and peer review. However, qualitative studies often run the risk of bias in spite of the fact that measures were implemented to reduce this effect. A small data sample also limits the ability to generalize the findings. Future studies could choose to include other religious denominations, expand the regional frame, increase the sample size, and incorporate quantitative measures to triangulate the data.

## Conclusion

DSEC is an intervention that is “short-term, stage-specific, needs-driven, and culturally responsive” (Schruba et al., 2018a, 2018b, p. 63). The findings of this study demonstrate that it can also be leveraged for long-term intervention in a period of extended crises. These priests, pastors, and their wives who served as DSEC practitioners in Puerto Rico were both rescuers and victims. In spite of the ongoing situation of stress and crises, positive psychological, social, and religious coping strategies were enacted to actively cope with the trauma. In the future, it will be important to assess the needs of these practitioners and to provide them with ongoing training and support.

## Data Availability

The data that support this research project can be made available upon reasonable request to the PI.

## Appendix 1

**Quote 1**: *Mi familia es del área metropolitana. Saber que ellos al mes tenían agua. En mi casa pasaron tres meses y no había agua. En enero ya tenían energía eléctrica y todavía en mi casa en enero no había energía eléctrica. Llegar de acá arriba y cruzar lo que yo, verdad, cruzar todo ese camino y llegar a mi casa, saber que la gente, ¿verdad?, la diferencia. La diferencia grande de estar viviendo en otros lugares. No tenían que cruzar, no tenían que cruzar esos lugares y podían llegar a su casa y tener energía eléctrica. Pero para nosotros no tan solo era cruzar el momento intenso, sino era llegar a la casa a las 7 u 8 de la noche, para entonces prender planta para que hubiese algo de energía, porque no llegábamos a descansar y nosotros, mi familia, en mi casa vivimos esa gran diferencia de como ya había cierto grado de normalidad en un sector de la isla y todavía nosotros estar viviendo de este, (señala a la ventana donde se escucha el tráfico muy ruidoso). Guiar desde tan… solo el guiar era asunto del diario vivir. Era asunto de diario vivir. Y esa diferencia, pues, fue muy marcada. Y lo vivimos, ¿verdad? O lo vimos, cómo todo corría y nosotros todavía estábamos en esa fatiga, en ese no terminar, en ese ajoro porque había gente que todavía no tenía nada, que volverían pero no tenían. Había trabajo, había trabajo pero no tenían cómo llegar a sus trabajos. No había manera, no había manera cómo cruzar. Acá arriba hubo caminos que literalmente se tardaron meses en abrirlos. Meses. Todavía hay una carretera, la 143, está cerrada porque esa carretera se fue. Se fue. Y se fue la carretera y se fue la mitad del monte. So una persona que vive 10 minutos de su trabajo tenía que todavía dar una inmensa vuelta para poder llegar porque el camino no está, porque la carretera no está y eso es real.* (P3).

**Quote 2:** *Todavía estábamos de la emergencia a emergencia. Ahí entonces cuando en enero decidí buscar y ….Necesitamos salir literalmente del país porque no había forma de desconectarse si no salíamos….Y creo que esos pocos espacios, esas vacaciones en enero nos ayudaron a respirar, nos ayudaron a recuperarnos, nos ayudaron a salir un poco y coger aire. Porque llega un momento que uno, más que cansado, se puede empezar a erosionar.* (P3).

**Quote 3**: *Pudimos tener un espacio de organizarnos y tener diferencias entre qué vamos a hacer, cómo lo vamos a hacer, pues ya llegamos a Puerto Rico como más fuerte. Pero si no fuera por eso.* (P2).

**Quote 4:** *Y entonces pues nos fuimos. Y de verdad fue buenísimo porque vinimos refrescados con una visión más clara de qué hacer, cómo hacer y ahí fue despejando todas las ideas… Inclusive quizás después de eso, al otro año, unos seis meses después de eso, …también nos separamos. Tuvimos como un retiro de tres días con diferentes pastores también amigo.* (P8).

**Quote 5:** *También suelo tomar retiros espirituales personales, suelo ir a casa de retiro que queda cerca del centro de la montaña y aprovecho unos días de descanso, de relajación y de contacto con Dios.* (P9).

**Quote 6:** *Obviamente nosotros ya llevamos 22 años pastoreando. Creo que la experiencia nos ayuda a que es importante descansar. Nosotros una vez inmediatamente empezaron a añadirse otros líderes, empezamos a tomar nuestros espacios. Empezamos a hacer muy celosos con nuestro tiempo de vacaciones para no caer en un burnout, porque en el pasado nos había pasado.* (P7).

**Quote 7**: *A través de la denominación tuvimos espacio. Tuvimos espacio. En lo personal teníamos espacio con profesionales de la salud porque uno tiene que tenerlo, porque uno se da cuenta que eso está ahí. Con todo y eso, ya había mencionado que al año, que al cumplir el año fue la primera vez lloré.* (P3).

**Quote 8:** *Y ese plan, en abril de este año, ellos a todos los pastores, ellos se pararon en el Hyatt de Manatí y ellos trajeron un recurso de afuera para decirles a los pastores… eh… cómo trabajar las depresiones, …cómo trabajar los momentos de crisis y tuvieron ese recurso de miércoles a miércoles para que los pastores de esta área pudieran llegar allí y poderse abrir con ese recurso y poder canalizar sus emociones.* (P5).

**Quote 9:** *Y nunca había vivido un temblor tan fuerte como aquí. Así que estos temblores me sembraron y gracias a Dios tuve que ir al psicólogo para que me sentara un poquito. Necesitamos la ayuda también de psicólogos. Y me ha servido mucho para seguir adelante en cuanto a este asunto personal.* (P10).

**Quote 10:** *Fue un tiempo bien fuerte, intenso, que llegó un momento en que dije: “Sabes que no puedo más, estoy cansada.” Incluso, verdad, comenzamos a recibir terapia.* (P4).

**Quote 11:** *Pero Dios está la fortaleza porque posiblemente tan pronto ocurre la necesidad de servir, la necesidad de hacer, se te hace que se te olvide lo demás. Que si estás cansado, que si todavía no te has recuperado del huracán. No, o sea cuando tú tienes que ayudar, cuando es una vocación de servicio, ¿verdad? Pues siempre, seguramente uno lo que hace es hacerlo lo mejor que uno pueda y se olvida de lo demás, ¿no?* (P9).

**Quote 12**: *Sí, las cosas que estábamos haciendo. Nosotros teníamos reuniones todas las semanas con* Christ Collaborative*, ¿verdad? Con las diferentes iglesias organizábamos cosas y sabíamos del proyecto. Así que la mente estaba distraída.* (P1).

**Quote 13**: *Bueno, obviamente lo más que me ayuda es la oración, ¿no? La oración diaria, la reflexión, el sacar tiempo para meditar, sacar tiempo para cerrar el celular y apagar todo y una vez es posible dar un paseo por la playa. La misa también diaria me ayuda mucho, ¿verdad?* (P9).

**Quote 14:** *Tengo amigos sacerdotes que nos reunimos a bebernos un café y a compartir, pero terminamos orando los unos por los otros. Y esos espacios son buenos.* (P5).

**Quote 15**: *Yo creo que yo estuve bien enfocado como pastor, líder… Toda mi vida he enseñado* leadership *liderado en misiones…..Trabajo, mi trabajo estaba centrada en la Iglesia, no en la comunidad. Mi vida cambió por completo desde el huracán y todavía sigue igual. Yo no creo que vaya a cambiar ya. María me enseñó que mi labor principal era con la comunidad, con la gente, que la Iglesia es llamado a ir por todo el mundo…Pues yo, en caso mío es personal. Mi vida cambió por completo. O sea, mi enfoque ahora es como trabajar dentro de la comunidad y la necesidad de la gente.* (P6).

**Quote 16**: *Entonces pues yo siento pues que yo tengo una vocación de servicio, ¿vedad?, y me ha puesto en una vocación de servicio a los más necesitados, ¿verdad?, por medio de mi trabajo. Por lo tanto, cuando se tuvo que activar, cuando nos tuvimos que activar, nos activamos.* (P9).

**Quote 17**: *Pero esos dos meses sí fueron una carga sobre mí y mi esposa, mi familia, total… De afuera como tal, pues fíjate, con el huracán como que hubo, hubo más ayuda de afuera. Quizás como la pandemia nos tocó a todos, pues como que no necesariamente yo recibí una llamada de alguien: “Mira. Ven para que tengas un tiempo de descanso.” O tal o cual cosa. Eso, en la pandemia eso no pasó. Yo creo que era que estábamos todos en el mismo, el mismo bote, ¿no?* (P7).

**Quote 18**: *En todo tiempo tenemos un grupo de apoyo de pastores y sacerdotes que nos reunimos cada cierto tiempo y podemos ventilar. Y eso, eso es una bendición. Así que yo como líder tenía esa red de apoyo. Pero no todo el mundo la tiene.* (P5).

**Quote 19**: *Y aún nosotros cuando salimos, ellos nos hicieron como, nos pastorearon, o sea, nos abrazaron, nos dieron como vamos a pasear por las comunidades de, nos hicieron entrevistar a la gente en la comunidad allá con gente que son indocumentados, pero que la iglesia, que es la iglesia que nos estaba llevando están ayudando. Querían que vieran otra cosa. Después fuimos con un pastor amigo a un evento que él tenía. Él nos quería salir, nos invitó a comer a otros sitios como para hablar, hablar de otro tema también. Y estuvimos con unos amigos que eran de la Iglesia Catacumba y otro de la organización Hunger, y eso para nosotros fue como lo suficientemente bueno como para regresar con muchas ganas de trabajar, más, más ganas de trabajar.* (P2).

**Quote 20**: *Estar consciente de que esa dualidad de que somos ayuda pero también somos damnificados. Y creo que eso fue muy importante, especialmente por decir los primeros dos meses que eran los momentos más caóticos. ¿Qué nosotros hacíamos? Bueno, nosotros dedicamos una hora antes de abrir nuestro servicio a rezar y hablar. Yo creo que era bien importante. Yo entendía que era vital para que nosotros pudiéramos llevar a cabo nuestra labor porque nadie puede dar de lo que no tiene. So primero uno se tiene que llenar para poder dar. Y yo creo que esa dinámica nos ayudó a todos porque, pues, la gente lloraba, la gente expresaba lo que estaba sucediendo en sus hogares.* (P9).

**Quote 21**: *Si algo yo no me sentí nunca descuidado fue en lo emocional y lo espiritual. Esa es la ventaja en trabajar con la Iglesia. Sabe, sí la Iglesia, que la gran mayoría estaban. Claro que parte fundamental de la Iglesia es trabajar en el área espiritual. Hubo momentos de desesperación.* (P6).

**Quote 22**: *Y yo, gracias a Dios que cuando yo llegué a esa parroquia, ya estaba el equipo de Cáritas trabajando. Se hizo un poquito mayor a raíz de las necesidades, pero gracias a Dios me rodea. Me rodea todo un equipo, todo un mundo de feligreses que me ayudan y que me alientan a seguir adelante. Sin ese equipo yo creo que me estaría atado a manos y pies y un mayor esfuerzo todavía para lograrlo.* (P10).

**Quote 23**: *Pero vimos la mano de Dios. En los vecinos de la iglesia, los líderes que ayudaron aquí. Sí, esto no fue un trabajo solo. No fue un trabajo de mucha gente, pero no fue un trabajo solo. Hubo gente que nos ayudó. Hubo gente que dio la milla extra. Hubo gente que nos recibió en su casa para atender misioneros que llegaron, misioneros que estuvieron en un espacio de una semana y nos daban nuevas fuerzas y por eso le damos gloria al Señor. Pero hubo mucha gente que llegó y si no hubiese sido eso, a uno vio la mano del Señor a través de la gente que llegó para levantarnos los brazos, para darnos nuevas fuerzas, nuevo aire y nuevo reposo. Así que fue un colectivo.* (P3).

**Quote 24**: *Tengo una esposa maravillosa, PI. (Se ríe) Una esposa maravillosa. Es trabajadora social. Así que hicimos un gran equipo y todo lo que aquí llega a base de distribuir de manera organizada, muy organizado, para poder proveer a la gente que tenía necesidad y urgencia para poder atender a aquellos que punto no tenían.* (P3).

**Quote 25**: *Hay mucho desconocimiento sobre el COVID y sobre todo lo demás. Y básicamente, pues, lo que hicimos fue comenzar a transmitir desde mi casa. Y yo tengo la bendición de que mis hijos todos son músicos, mi esposa. Así que básicamente estábamos corriendo todo en ese proceso tecnológico.* (P7).

**Quote 26:** *Algo que nos ayuda a nosotros es que P2, mi esposa, ella ha trabajado en el tema de social media por tantos años y nosotros teníamos equipo para grabar. Por ejemplo, yo llevo usando Zoom hace como siete años. Así que es una herramienta que no la tuve que descubrir.* (P1).

**Quote 27:** *Es difícil ahora. Porque dependiendo en donde están ubicado. Por ejemplo, yo sé que en el caso de P3, que lo conoces, a él se le hizo difícil de que la potencia del internet en la zona donde él está ubicado es más lenta, más no tenía equipo. Y la iglesia donde asiste la mamá de* [un empleado] *en Ciales, ellos no hacían nada que no fuera* voice messages *por WhatsApp.* (P1).
